# Real-world evaluation of CDK4/6 inhibitors in hormone receptor-positive metastatic breast cancer: prognostic effect of proton pump inhibitor use

**DOI:** 10.1093/oncolo/oyaf268

**Published:** 2025-09-02

**Authors:** Bo-Fang Chen, Jiun-I Lai, Yi-Fang Tsai, Pei-Ju Lien, Yen-Shu Lin, Chin-Jung Feng, Yen-Jen Chen, Han-Fang Cheng, Ta-Chung Chao, Chun-Yu Liu, Ling-Ming Tseng, Chi-Cheng Huang

**Affiliations:** Division of Breast Surgery, Department of Surgery, Taipei Veterans General Hospital, Taipei 112, Taiwan; Comprehensive Breast Health Center, Department of Surgery, Taipei Veterans General Hospital, Taipei 112, Taiwan; School of Medicine, National Yang-Ming Chiao-Tung University, Taipei 112, Taiwan; Division of Medical Oncology, Department of Oncology, Taipei Veterans General Hospital, Taipei 112, Taiwan; Center of Immuno-Oncology, Department of Oncology, Taipei Veterans General Hospital, Taipei 112, Taiwan; Institute of Clinical Medicine, School of Medicine, National Yang-Ming Chiao-Tung University, Taipei 112, Taiwan; Division of Breast Surgery, Department of Surgery, Taipei Veterans General Hospital, Taipei 112, Taiwan; Comprehensive Breast Health Center, Department of Surgery, Taipei Veterans General Hospital, Taipei 112, Taiwan; School of Medicine, National Yang-Ming Chiao-Tung University, Taipei 112, Taiwan; Comprehensive Breast Health Center, Department of Surgery, Taipei Veterans General Hospital, Taipei 112, Taiwan; Division of Breast Surgery, Department of Surgery, Taipei Veterans General Hospital, Taipei 112, Taiwan; Comprehensive Breast Health Center, Department of Surgery, Taipei Veterans General Hospital, Taipei 112, Taiwan; School of Medicine, National Yang-Ming Chiao-Tung University, Taipei 112, Taiwan; Comprehensive Breast Health Center, Department of Surgery, Taipei Veterans General Hospital, Taipei 112, Taiwan; School of Medicine, National Yang-Ming Chiao-Tung University, Taipei 112, Taiwan; Division of Plastic and Reconstructive Surgery, Department of Surgery, Taipei Veterans General Hospital, Taipei 112, Taiwan; Division of Breast Surgery, Department of Surgery, Taipei Veterans General Hospital, Taipei 112, Taiwan; Comprehensive Breast Health Center, Department of Surgery, Taipei Veterans General Hospital, Taipei 112, Taiwan; School of Medicine, National Yang-Ming Chiao-Tung University, Taipei 112, Taiwan; Division of Breast Surgery, Department of Surgery, Taipei Veterans General Hospital, Taipei 112, Taiwan; Comprehensive Breast Health Center, Department of Surgery, Taipei Veterans General Hospital, Taipei 112, Taiwan; School of Medicine, National Yang-Ming Chiao-Tung University, Taipei 112, Taiwan; Comprehensive Breast Health Center, Department of Surgery, Taipei Veterans General Hospital, Taipei 112, Taiwan; School of Medicine, National Yang-Ming Chiao-Tung University, Taipei 112, Taiwan; Division of Cancer Prevention, Department of Oncology, Taipei Veterans General Hospital, Taipei 112, Taiwan; Comprehensive Breast Health Center, Department of Surgery, Taipei Veterans General Hospital, Taipei 112, Taiwan; School of Medicine, National Yang-Ming Chiao-Tung University, Taipei 112, Taiwan; Division of Medical Oncology, Department of Oncology, Taipei Veterans General Hospital, Taipei 112, Taiwan; Division of Breast Surgery, Department of Surgery, Taipei Veterans General Hospital, Taipei 112, Taiwan; Comprehensive Breast Health Center, Department of Surgery, Taipei Veterans General Hospital, Taipei 112, Taiwan; School of Medicine, National Yang-Ming Chiao-Tung University, Taipei 112, Taiwan; Department of Surgery, Taipei Veterans General Hospital, Taipei, 112 Taiwan; Division of Breast Surgery, Department of Surgery, Taipei Veterans General Hospital, Taipei 112, Taiwan; Comprehensive Breast Health Center, Department of Surgery, Taipei Veterans General Hospital, Taipei 112, Taiwan; Institute of epidemiology and preventive medicine, School of Public Health, College of Public Health, National Taiwan University, Taipei 100, Taiwan

**Keywords:** CDK4/6 inhibitor, breast cancer, real-world data, proton pump inhibitor, treatment outcomes

## Abstract

**Importance:**

This study highlights the potential negative impact of the co-administration of proton pump inhibitors (PPI) on treatment outcomes in patients with hormone receptor-positive (HR+) metastatic and recurrent breast cancer who were receiving CDK4/6 inhibitor (CDK4/6i) treatment, providing real-world evidence to guide treatment strategies.

**Objective:**

To determine whether the co-administration of proton pump inhibitors (PPI) affects treatment outcomes, including progression-free survival (PFS) and overall survival (OS), in patients with HR+ metastatic and recurrent breast cancer receiving CDK4/6i treatment.

**Design**

This was a retrospective analysis.

**Setting**

The study was conducted at a single medical institution in Taipei, Taiwan.

**Participants**

The study included 359 patients with HR+ metastasis and relapse breast cancer treated between 2018 and 2023.

**Intervention(s) or Exposure(s)**

Patients were grouped into those receiving CDK4/6i plus endocrine therapy without any antacid medications, and those with a combination of either a PPI or H2 receptor blocker.

**Main Outcome(s) and Measure(s)**

The main outcomes measured were PFS and OS.

**Results:**

There were 55.7% patients in the no-antacid group, 33.1% in the PPI group, and 11.1% in the H2-blocker only group. Patients receiving PPI in combination with CDK4/6i and endocrine therapy had significantly shorter PFS (hazard ratio [HR] = 2.298, *P* < 0.001) and OS (HR = 3.03, *P* < 0.001). The H2 blocker group also showed a trend toward poorer PFS (HR = 1.987, *P* < 0.001) and OS compared with those without antacid use (HR = 3.380, *P* = 0.226). These trends were shown in both the overall cohort and first-line treatment, regardless of the specific CDK4/6i used. No significant differences were observed between types of PPIs. Additionally, increased PPI usage time proportion during CDK4/6i treatment was associated with a higher risk of disease progression and mortality.

**Conclusion and Relevance:**

The use of PPIs and H2 blockers, in combination with CDK4/6i, was associated with adverse effects on PFS and OS in patients with HR+ metastatic or recurrent breast cancer in a real-world setting. Clinicians should exercise caution when prescribing PPIs to patients undergoing CDK4/6i therapy.

**Key Points:**

- In this retrospective analysis of 359 patients, the co-administration of PPIs with CDK4/6i was associated with significantly shorter PFS and OS.
- Clinicians should be cautious when prescribing PPIs to patients receiving CDK4/6i therapy due to the potential for adverse effects on treatment outcomes.

Implications for Practice This study highlights the potential negative impact of co-administering PPIs or H2 blockers with CDK4/6 inhibitors in HR+ metastatic and recurrent breast cancer. Clinicians should carefully evaluate the necessity of acid-suppressive therapy during CDK4/6i treatment, as PPI use is associated with significantly worse progression-free and overall survival. These findings underscore the importance of medication reconciliation and patient education to avoid unnecessary PPI use. When acid suppression is essential, alternative strategies should be considered.

## Introduction

The advent of cyclin-dependent kinase 4/6 inhibitors (CDK4/6i), including palbociclib, ribociclib, and abemaciclib, has marked a substantial advancement in the treatment paradigm for hormone receptor-positive (HR+), HER2-negative metastatic breast cancer.^1–3^ However, the efficacy of orally administered CDK4/6 inhibitor treatment can be influenced by a variety of factors.

The primary indication for antacid prescription in cancer patients appears to be the relief of gastrointestinal symptoms. The prevalence of antacid use, particularly proton pump inhibitors (PPIs), among cancer patients is substantial, with initial estimates suggesting a range of 20%-55%. This significant proportion of the oncology population utilizing acid-reducing medications necessitates a thorough understanding of potential interactions with other commonly prescribed cancer therapies.^4^^,^^5^ PPIs exert their therapeutic effect through irreversibly binding to the hydrogen/potassium adenosine triphosphatase (H+/K+-ATPase) enzyme, which is commonly referred to as the proton pump. Its inhibition directly and potently suppresses gastric acid production.^6^ This PPI-induced elevation of gastric pH can have a detrimental effect on the dissolution of weakly basic anticancer drugs in the stomach. Inadequate dissolution may result in decreased absorption, which causes lower overall bioavailability of the anticancer drug, potentially leading to subtherapeutic plasma concentrations and a diminished therapeutic effect.^7^

Nevertheless, the superfamily cytochrome P450 (CYP450) enzyme system is primarily located in the liver and plays a central role in drug metabolism machinery. The CYP450 system is involved in the metabolism of both PPIs and CDK4/6i, making it essential to examine the potential for drug–drug interactions at this metabolic level.^8–11^ The competition of two drugs binding to the same enzyme’s active site can result in decreased metabolism and consequently, increased plasma concentrations of one or both drugs, leading to an elevated risk of adverse effects or altering the intended efficacy of the medication.

Even though several anticancer drugs, such as capecitabine and Pazopanib, have been proven to have diminished effectiveness when co-administered with PPIs, current clinical guidelines on the co-administration of acid-reducing therapies with anticancer agents are limited and sometimes conflicting.^12^^,^^13^ Therefore, a need for more definitive research to guide clinical practice and optimize treatment outcomes for patients with HR+, HER2− metastatic breast cancer receiving CDK4/6i is crucial. This dissertation aims to evaluate the prognostic impact of the co-administration of proton pump inhibitors on treatment outcomes in patients with HR+ metastatic and recurrent breast cancer who were receiving CDK4/6i treatment, providing real-world evidence to guide treatment strategies.

## Methods

### Study population

This observational, retrospective study enrolled patients diagnosed with HR+ metastatic or recurrent breast cancer who received treatment with CDK4/6i (palbociclib, ribociclib, or abemaciclib) between January 2019 and December 2023 at Taipei Veterans General Hospital, a tertiary referral medical center in Northern Taiwan. Patients who received CDK4/6i therapy for ≤14 days or those with other malignancies or early-stage breast cancer were excluded from the study.

Patients prescribed any PPI, including lansoprazole, rabeprazole, pantoprazole, or esomeprazole, for a duration exceeding one day within the CDK4/6i treatment period were classified into the PPI group. Furthermore, patients who received any histamine-2 receptor antagonist (H2-blocker), including famotidine, ranitidine, or cimetidine, for more than one day but did not receive PPI treatment during the CDK4/6i treatment period were defined as the H2-blocker group. Patients who did not receive any PPI or H2-blocker treatment were classified as the no-antacid group.

Clinical treatment protocols followed the guidelines established by the Comprehensive Breast Health Center at Taipei Veterans General Hospital, which are based on recommendations from the National Comprehensive Cancer Network (NCCN), the European Society for Medical Oncology (ESMO), and the St Gallen International Consensus Conference guidelines.^14–16^ Clinicopathological data, disease status, prescription details, and adverse events were extracted from the Big Data Center and electronic medical records of Taipei Veterans General Hospital. To ensure patient confidentiality, all potentially identifying information will be encrypted and anonymized, including the removal of names, addresses, and other identifiable details. The study protocol was approved by the Institutional Review Board of Taipei Veterans General Hospital (IRB-TPEVGH No.: 2024-04-014AC) with a waiver of informed consent.

### Data collections and outcomes

HR-positive breast cancer was defined by positive estrogen receptor (ER) status, indicated by ≥1% of tumor cells exhibiting nuclear staining via immunohistochemistry (IHC) assay. Patients with Human Epidermal Growth Factor Receptor 2 (HER2) test results of IHC 3+ (positive) or 2+ (equivocal) with subsequent confirmation of gene amplification by fluorescence in situ hybridization (FISH) were classified as HER2-positive; otherwise, they were considered HER2-negative.^17^ Pathology results were determined using the most recent available data, which could originate from the primary breast tumor, local recurrence, or metastatic lesions. Adverse events (AEs) were documented by treating physicians or extracted from the institutional Big Data Center, utilizing the Common Terminology Criteria for Adverse Events (CTCAE) version 5.0.^18^ CDK4/6i dose reduction was defined as the administration of a continuously lower dose than the standard dosage on more than two separate occasions. The duration of PPI and H2-blocker prescription coverage during the CDK4/6i treatment period was defined as the “antacid use proportion in CDK4/6i.” Similarly, the duration of PPI prescription coverage during the CDK4/6i treatment period was defined as the “PPI use proportion in CDK4/6i.” The Charlson Comorbidity Index (CCI) was employed to quantify the burden of patients’ medical comorbidities, with higher CCI scores indicating increased mortality risk.^19^ CCI data were derived from International Classification of Diseases, Ninth and Tenth Revision (ICD-9 and ICD-10) codes documented in inpatient records with relevant diagnostic codes or from more than two outpatient diagnoses with related codes. The total CCI score represents the sum of weighted values assigned to individual comorbid conditions. Progression-free survival (PFS) was defined as the time from the initiation of CDK4/6 inhibitor treatment to the first documented occurrence of disease progression (local recurrence or distant metastasis) or death from any cause, whichever occurred earliest. Overall survival (OS) was defined as the time from the initiation of CDK4/6 inhibitor treatment until death from any cause. For both endpoints, patients without an event were censored at the date of their last follow-up.

### Statistical analysis

All clinicopathological characteristics and CDK4/6i treatment conditions were compared across the no-antacid, PPI, and H2-blocker groups using the chi-squared (χ^2^) test, analysis of variance (ANOVA), or Fisher’s exact test, as appropriate. Bonferroni correction was applied to adjust for multiple comparisons. PFS and OS between the groups was assessed using Kaplan–Meier survival curve analysis, and statistical significance was determined using the log-rank test. Furthermore, the Cox proportional hazards model was employed to estimate the hazard ratio for relapse. Statistical significance was defined as a two-sided *P*-value of less than 0.05 for all analyses. All statistical analyses were performed using R statistical software, version 4.4.2.^20^

## Results

### Study population and patient characteristics

The study cohort comprised 359 patients, distributed as follows: 200 (55.7%) in the no-antacid group, 119 (33.1%) in the PPI group, and 40 (11.1%) in the H2-blocker group. Within the H2-blocker group, 60 patients received famotidine, 11 received ranitidine, and 4 received cimetidine. In the PPI group, the distribution was 40 patients treated with esomeprazole, 29 with lansoprazole, 29 with rabeprazole, and 21 with pantoprazole. The median follow-up duration for the entire cohort was 28.0 months (no-antacid group: 29.8 months; PPI group: 24.9 months; H2-blocker group: 24.7 months), with no statistically significant difference observed between the groups (*P* = 0.325). In Table 1, there were no significant differences between the three groups for patient age, breast cancer histology, pathological status, or disease status. The distribution of metastasis sites, including bone, liver, lung, distant lymph nodes, and other locations, also did not differ significantly between the groups. However, the PPI group exhibited a significantly higher proportion of patients with brain metastasis (10.1%) compared with both the no-antacid group (3.5%) and the H2-blocker group (0%) (*P* < 0.05). Regarding the CCI, the PPI group had 42.0% of patients with a score ≤ 8, 31.9% with a score of 8-10, and 26.1% with a score > 10. This distribution was similar to the H2-blocker group (45%, 27.5%, and 27.5%, respectively), but both the PPI and H2-blocker groups had significantly higher CCI scores compared with the no-antacid group (64%, 24%, and 12%, respectively).

**Table 1.** Clinicopathological characteristics based on no antacids use, co-administration of proton pump inhibitors, and H2-blocker only (*N* = 359).

Characteristics	No antacids *n* = 200 (%)	PPI *n* = 119 (%)	H2 blocker only *n* = 40 (%)	*P* value
**Age (mean ± SD)**	60 ± 12.6	59 ± 11.9	64 ± 11.5	.319
** <50**	39 (19.5)	29 (24.4)	3 (7.5)	
** 50-70**	111 (55.5)	64 (53.8)	23 (57.5)	
** ≥70**	50 (25.0)	26 (21.8)	14 (35.0)	
**Histology**				
** IDC**	158 (79.0)	98 (82.4)	32 (80.0)	.263
** ILC**	14 (7.0)	9 (7.6)	6 (15.0)	
** Other/unknown**	28 (14.0)	12 (10.1)	2 (5.0)	
**Pathologic status**				
** ER+**	199 (99.5)	119 (100)	40 (100)	1
** PR+**	169 (84.5)	93 (78.2)	34 (85.0)	.331
** HER2−**	193 (96.5)	110 (92.4)	40 (100.0)	.190
** Ki ≥20**	86 (43.0)	54 (45.4)	25 (62.5)	.047
**Disease status**				
** De novo stage IV**	75 (37.5)	36 (30.3)	15 (37.5)	.416
** Distant metastasis**	183 (91.5)	115 (96.6)	36 (90.0)	.140
**Visceral metastasis**	138 (69.0)	89 (74.8)	28 (70.0)	.559
**Bone only**	40 (20)	16 (13.4)	7 (17.5)	.324
**Site of metastasis**				
** Bone**	118 (59.0)	83 (69.7)	26 (65.0)	.152
** Liver**	54 (27.0)	40 (33.6)	12 (30.0)	.455
** Lung**	94 (47.0)	54 (45.4)	19 (47.5)	.953
** Brain**	7 (3.5)	12 (10.1)	0 (0)	.015^a^
** Dist. LN and others**	31 (15.5)	28 (23.5)	5 (12.5)	.137
**CCI**				
** ≤8**	128 (64.0)	50 (42.0)	18 (45.0)	.001^b^
** 8-10**	48 (24.0)	38 (31.9)	11 (27.5)	
** >10**	24 (12.0)	31 (26.1)	11 (27.5)	

Abbreviations: Dist.: distant, LN: lymph nodes.

a Brain: No vs PPI: 0.0254; PPI vs H2: 0.0381; no vs H2: 0.6043.

b Charlson Comorbidity Index: Bonferroni correction *P* value: No antacids vs PPI: *P* = .001; no antacid vs H2-blocker: *P* = .074; PPI vs H2-blocker: *P* = 1.0000.

### CDK4/6 inhibitor treatment strategies

Table 2 presents the CDK4/6i treatment strategies across the three patient groups. The mean follow-up duration was 32.3 months in the no-antacid group, 27.1 months in the PPI group, and 26.8 months in the H2-blocker group. The distribution of CDK4/6i type and concurrent endocrine therapy was balanced across the groups. First-line CDK4/6i treatment constituted the majority in all groups (no antacid: 81.5%; PPI: 73.9%; H2-blocker: 82.5%). The proportion of patients who received prior chemotherapy was also comparable across the groups (no antacid: 17.5%; PPI: 20.2%; H2-blocker: 15%). The median duration of PPI and H2-blocker prescription coverage during the CDK4/6i treatment period was 20% of the days in both groups. The dose reduction rates for CDK4/6i were 25% in the no-antacid group, 24.4% in the PPI group, and 30% in the H2-blocker group, with no statistically significant difference observed. However, the PPI group exhibited a significantly higher incidence of grade 3 neutropenia (56.3%) and an increase of at least 1.5 times the baseline serum creatinine (64.7%) compared with the no-antacid group (42% and 48%) (*P* < 0.05).

**Table 2.** The CDK4/6 inhibitors treatment strategies and the prevalence of adverse effects.

	No antacids *n* = 200 (%)	PPI *n* = 119 (%)	H2 blocker only *n* = 40 (%)	*P* Value
**Follow up times (days)**	905.5 [20-1985]	758 [60-2159]	750 [50-2128]	.325
**CDK4/6i agent**				
** Palbociclib**	88 (44.0)	60 (50.4)	19 (47.5)	.622
** Ribociclib**	95 (47.5)	46 (38.6)	17 (42.5)	
** Abemaciclib**	17 (8.5)	13 (10.9)	4 (10.0)	
**CDK4/6i line**				
** First line**	163 (81.5)	88 (73.9)	33 (82.5)	.447
** Second line**	9 (4.5)	10 (8.4)	1 (2.5)	
** ≥Third line**	28 (14.0)	21 (17.6)	6 (15.0)	
**CDK4/6i use time (median, days)**	615 [18-1985]	313 [21-1965]	430 [49-2026]	<.001^a^
**Antacid use time (median, days)**	-	71 [1-758]	46 [3-761]	.253
**Antacids use proportion in CDK4/6i**	-	20.0% [0.3-100]	20.4% [0.2-100]	.155
**Endocrine agent (first line)**				
** Letrozole**	127 (63.5)	74 (62.2)	23 (57.5)	.347
** Fulvestrant**	28 (14.0)	19 (16.0)	3 (7.5)	
** Exemestane**	24 (12.0)	10 (8.4)	6 (15.0)	
** Anastrozole**	6 (3.0)	6 (5.0)	5 (12.5)	
** Tamoxifen**	15 (7.5)	9 (7.6)	3 (7.5)	
**Previous chemotherapy**	35 (17.5)	24 (20.2)	6 (15.0)	.765
**Dose reduction**	50 (25.0)	29 (24.4)	12 (30.0)	.773
**Neutropenia ≥G3**	84 (42.0)	67 (56.3)	24 (60.0)	.016^b^
**Cr increased ≥1.5 times baseline**	96 (48.0)	77 (64.7)	22 (55.0)	.015^c^

Abbreviations: G3: grade 3; Cr: creatinine.

a PPI to No: <0.0001, H2 to no: <0.001, H2 to PPI: <0.001.

b PPI to No: 0.015, H2 to no: 0.055, H2 to PPI: 0.716.

c PPI to No: 0.005, H2 to no: 0.489, H2 to PPI: 0.346.

### Disease relapse and mortality

**Figure 1.**
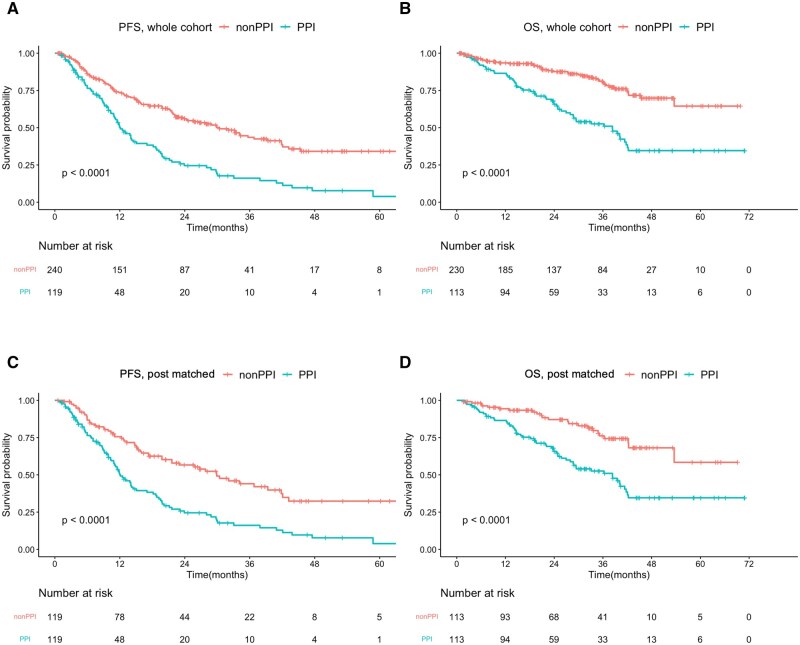
Kaplan–Meier survival curve analyses for patients treated with CDK4/6 inhibitors with and without PPIs. (A) PFS and (B) OS in the overall cohort. (C) PFS and (D) OS after propensity score matching.

The PFS of the entire cohort is illustrated in Figure 1A. Patients who did not receive PPIs demonstrated significantly superior PFS compared with those who received PPIs in combination with CDK4/6i treatment. The median PFS (mPFS) was 30.4 months in the non-PPI group and 12.1 months in the PPI group, with a hazard ratio (HR) of 2.298 (95% confidence interval [CI], 1.733-3.046; *P* < 0.05). The median overall survival (mOS) was 38.3 months in the PPI group, but not reached in the non-PPI group, with an HR of 3.03 (95% CI, 2.033-4.517; *P* < 0.05) (Figure 1B). Given the significant differences in baseline patient characteristics, specifically a higher CCI and brain metastasis rate in the PPI group, propensity score matching was performed based on CCI score and the presence of brain metastasis. Following matching, the PFS remained significantly different between the non-PPI and PPI groups (29.9 months vs. 12.1 months, respectively; HR 2.279, 95% CI, 1.629-3.181; *P* < 0.05) (Figure 1C). The mOS was not reached in the post-matched non-PPI group, while the PPI group had an mOS of 38.3 months (HR 2.75, 95% CI, 1.693-4.465; *P* < 0.05) (Figure 1D).

**Figure 2.**
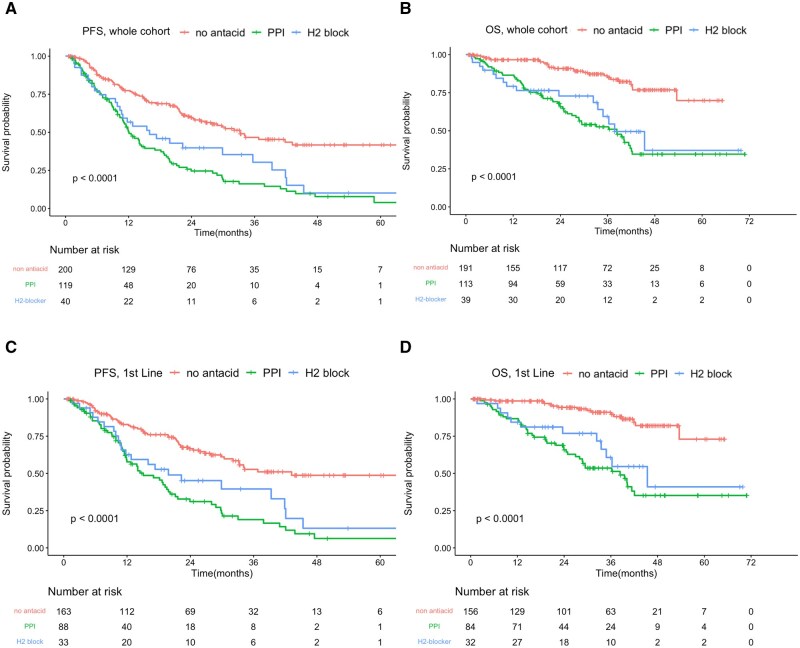
Kaplan–Meier survival curve analyses for PFS and OS among patients treated with CDK4/6 inhibitors without antacids, with PPIs, or with H2 blockers only. (A) PFS and (B) OS in the overall cohort. (C) PFS and (D) OS in patients receiving CDK4/6 inhibitor as first-line treatment.

Considering the potential influence of national health insurance policies and financial constraints leading to a higher prescription rate of H2-blockers over PPIs for patients with gastrointestinal symptoms, the non-PPI group was further stratified into the no-antacid and H2-blocker (without prescription of PPI) groups. Consistent with the previous findings, significant differences in PFS and OS were observed across these three groups. The mPFS was 33.3 months in the no-antacid group, 16.0 months in the H2-blocker group (HR 1.987, 95% CI, 1.30-3.031; *P* < 0.05), and 12.1 months in the PPI group (HR 2.637, 95% CI, 1.949-3.568; *P* < 0.05) (Figure 2A). The mOS was not reached in the no-antacid group, while it was 37.8 months in the H2-blocker group (HR 3.380, 95% CI, 1.813-6.303; *P* < 0.05) and 38.3 months in the PPI group (HR 4.144, 95% CI, 2.604-6.593; *P* < 0.05) (Figure 2B).

Figure 2C and D illustrate the outcomes for cohorts treated with CDK4/6i as first-line therapy. The mPFS was 43.2 months in the no-antacid group, 19.8 months in the H2-blocker group (HR 2.105, 95% CI, 1.288-3.441; *P* < 0.05), and 15.1 months in the PPI group (HR 2.900, 95% CI, 2.015-4.173; *P* < 0.05). Similar trends were observed for mOS: 45.36 months in the H2-blocker-only group (HR 3.97, 95% CI, 1.877-8.396; *P* < 0.05), and 38.37 months in the PPI group (HR 5.746, 95% CI, 3.226-10.234; *P* < 0.05). The mOS was not reached in the no-antacid group.

Comparing the non-PPI group (mPFS 30.42 months) with individual PPI types revealed significant differences in PFS (*P* < 0.05) for lansoprazole (mPFS 11.95 months, HR 2.383, 95% CI, 1.438-3.950), rabeprazole (mPFS 20.17 months, HR 1.750, 95% CI, 1.098-2.790), pantoprazole (mPFS 14.16 months, HR 2.417, 95% CI, 1.424-4.101), and esomeprazole (mPFS 10.28 months, HR 2.764, 95% CI, 1.861-4.106) (Figure S1A, see online supplementary material for a color version of this figure). For mOS, only rabeprazole did not reach the median and showed no significant difference compared with the non-PPI group (HR 0.898, 95% CI, 0.355-2.272; *P* = 0.821). In contrast, lansoprazole (mOS 39.48 months, HR 3.188, 95% CI, 1.7661-5.754), pantoprazole (mOS 36.24 months, HR 4.087, 95% CI, 2.089-7.996), and esomeprazole (mOS 23.92 months, HR 4.485, 95% CI, 2.746-7.324) were associated with significantly shorter mOS (Figure S1B, see online supplementary material for a color version of this figure).

**Figure 3.**
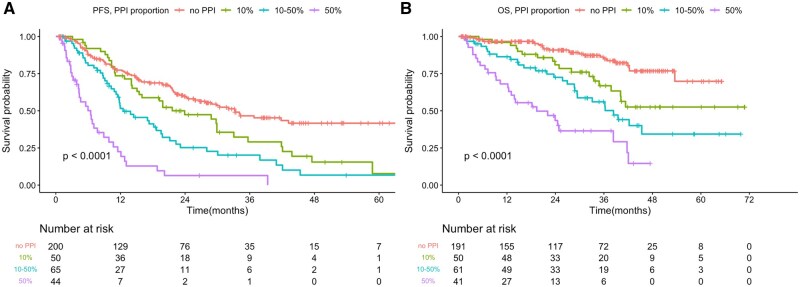
Kaplan–Meier survival curve analyses for PFS (A) and OS (B) among patients treated with CDK4/6 inhibitors without antacids and with proportion of PPI usage.

Regarding the proportion of PPI usage time during the CDK4/6i treatment period, a higher proportion of PPI usage was significantly associated with poorer mPFS and mOS (all *P* < 0.05) (Figure 3). Compared with the non-PPI group (mPFS 33.28 months), patients with PPI usage less than 10% of the CDK4/6i treatment duration had an mPFS of 23.92 months (HR 1.568, 95% CI, 1.061-2.318). Those with 10%-50% PPI usage had an mPFS of 12.48 months (HR 2.451, 95% CI, 1.706-3.520), and patients with more than 50% PPI usage had an mPFS of only 6.47 months (HR 6.545, 95% CI, 4.330-9.893). The mOS was not reached in patients with less than 10% PPI usage (HR 2.336, 95% CI, 1.281-4.262), while those with 10%-50% PPI usage had an mOS of 38.37 months (HR 3.765, 95% CI, 2.206-6.424), and those with more than 50% PPI usage had an mOS of only 19.38 months (HR 8.309, 95% CI, 4.817-14.331).

Notably, the poorer prognosis associated with PPI and H2-blocker usage was also observed in patients treated with both palbociclib and ribociclib. In the palbociclib cohort, the mPFS was 33.21 months in the no-antacid group, but only 15.47 months in the H2-blocker group (HR 2.393, 95% CI, 1.315-4.356) and 11.96 months in the PPI group (HR 2.924, 95% CI, 1.898-4.506). The mOS was not reached in either the H2-blocker or the no-antacid group (*P* = 0.133), while the PPI group had a significantly shorter mOS of 40.11 months (HR 4.780, 95% CI, 2.3907-9.558; *P* < 0.05) (Figure S2A and B, see online supplementary material for a color version of this figure). Regarding ribociclib, the no-antacid group had an mPFS of 34.2 months, the H2-blocker group had a slightly lower mPFS of 29.9 months (HR 1.519, 95% CI, 0.772-2.989; *P* = 0.227), and the PPI group had a significantly shorter mPFS of only 14.2 months (HR 2.409, 95% CI, 1.504-3.860; *P* < 0.05) (Figure S2C and D, see online supplementary material for a color version of this figure). In the abemaciclib cohort, however, no significant differences in mPFS and mOS were observed, likely due to the limited sample size (Figure S2E and F, see online supplementary material for a color version of this figure).

Table 3 showed univariate Cox regression analysis which identified HER2 IHC status, Ki-67 index, visceral metastasis, liver metastasis, prior chemotherapy, CDK4/6i treatment line, PPI usage (any vs. none), PPI use proportion in CDK4/6i, and CCI as significant risk factors for disease relapse. Conversely, PR status, de novo stage IV disease, and bone-only metastasis were associated with a significantly decreased risk of progression. Multivariate Cox regression analysis revealed that only Ki-67 index, liver metastasis, and PPI use proportion in CDK4/6i remained significant independent risk factors for disease progression. Regarding overall mortality, univariate Cox regression analysis demonstrated that ILC histology, Ki-67 index, visceral metastasis, liver metastasis, prior chemotherapy, CDK4/6i treatment line, PPI usage (any vs. none), PPI use proportion, and CCI were significant risk factors for mortality. Bone-only metastasis was the only factor associated with a decreased risk of mortality in the univariate analysis. In the multivariate Cox regression model, only Ki-67 index, visceral metastasis, PPI use proportion, and CCI emerged as significant independent risk factors for overall mortality (all *P* < 0.05) (Table 4).

**Table 3.** Risk factors of disease progression in breast cancer patients treated with CDK4/6 inhibitors using the univariate and multivariate Cox proportional hazards model.

	Univariate analysis			Multivariate analysis		
Risk factor	Hazards ratio	95% Confidence Interval	*P*-value	Hazards ratio	95% Confidence interval	*P*-value
**Age**	0.997	0.986-1.009	.610			
**Histology**						
** IDC**	1					
** ILC**	1.131	0.677-1.891	.638			
** other**	0.678	0.417-1.104	.118			
**PR status**	0.995	0.992-0.999	.023*	0.998	0.993-1.003	.393
**HER2 (IHC)**	1.214	1.006-1.465	.043*	0.905	0.689-1.189	.472
**Ki-67**	1.028	1.018-1.038	<.001*	1.030	1.020-1.040	<.001*
***De novo* stage IV**	0.733	0.542-0.990	.043*	0.687	0.475-0.992	.045*
**Metastasis site**						
** Visceral metastasis**	1.403	1.016-1.937	.040*	1.202	0.697-2.071	.508
** Bone only metastasis**	0.615	0.417-0.907	.014*	0.868	0.475-1.586	.646
** Liver metastasis**	2.638	1.977-3.519	<.001*	2.030	1.308-3.150	.002*
** Lung metastasis**	0.911	0.689-1.204	.513			
** Brain metastasis**	0.961	0.523-1.767	.899			
**Prior chemotherapy**	2.363	1.710-3.266	<.001	1.433	0.871-2.360	.157
**CDK4/6i agent**						
** palbociclib**	1					
** ribociclib**	0.883	0.659-1.184	.405			
** abemaciclib**	1.548	0.944-2.539	.084			
**CDK4/6i line**	1.186	1.117-1.259	<.001*			
**PPI use**	2.298	1.733-3.046	<.001*			
**PPI use proportion in CDK4/6i**	1.025	1.020-1.029	<.001*	1.022	1.015-1.029	<.001*
** 0%**	1					
** 10%**	1.470	1.001-2.158	.0493*			
** 10-50%**	2.569	1.781-3.705	<.001*			
** >50%**	6.537	4.321-9.890	<.001*			
**H2-blocker use only**	1.271	0.862-1.874	.226			
**CCI**	1.367	1.073-1.742	.011*	1.343	0.975-1.851	.071

* *P < *.05.

**Table 4.** Risk factors of mortality in breast cancer patients treated with CDK4/6 inhibitors using the univariate and multivariate Cox proportional hazards model.

	Univariate analysis			Multivariate analysis		
Risk factor	Hazard ratio	95% Confidence interval	*P*-value	Hazards ratio	95% Confidence interval	*P*-value
**Age**	1.006	0.989-1.023	0.51			
**Histology**						
** IDC**	1					
** ILC**	2.067	1.124-3.800	.019*			
** other**	0.617	0.298-1.278	.194			
**Progesterone receptor status**	0.997	0.991-1.002	.257			
**HER2 (IHC)**	1.152	0.877-1.512	.309			
**Ki-67**	1.026	1.013-1.039	<.001*	1.024	1.011-1.037	<.001*
***De novo* stage IV**	1.254	0.838-1.878	.271			
**Metastasis site**						
** Visceral metastasis**	2.159	1.281-3.641	.004*	2.513	1.065-5.930	.035*
** Bone only metastasis**	0.426	0.221-0.820	.011*	1.375	0.506-3.740	.533
** Liver metastasis**	2.815	1.89-4.194	<.001*	1.412	0.814-2.450	.220
** Lung metastasis**	0.9212	0.620-1.369	.685			
** Brain metastasis**	1.738	0.876-3.451	.114			
**Prior chemotherapy**	2.196	1.41-3.42	<.001*	1.301	0.692-2.444	.414
**CDK4/6i agent**						
** Palbociclib**	1					
** Ribociclib**	1.022	0.672-1.552	.920			
** Abemaciclib**	1.916	0.982-3.739	.057			
**CDK4/6i line**	1.196	1.108-1.291	<.001*			
**PPI use**	3.03	2.033-4.517	<.001*			
**PPI use proportion in CDK4/6i**	1.021	1.016-1.026	<.001*	1.016	1.009-1.023	<.001*
** 0**	1					
** 10%**	2.270	1.266-4.069	.006*			
** 10-50%**	3.591	2.084-6.188	<.001*			
** >50%**	8.108	4.704-13.976	<.001*			
**H2-blocker use only**	1.437	0.841-2.455	.185			
**CCI**	1.722	1.210-2.449	.003*	1.808	1.115-2.932	.016*

* *P < *.05.

## Discussion

The introduction of CDK4/6i has revolutionized the treatment landscape for HR+, HER2− metastatic breast cancer. However, the frequent co-prescription of PPIs in this patient population raises important questions about potential drug–drug interactions and their impact on treatment outcomes

A retrospective study by Del Re et al. found that concurrent PPI use significantly reduced PFS in patients receiving palbociclib as first-line therapy (14.0 months vs 37.9 months, *P* < 0.0001).^21^ However, another retrospective study by Takahashi et al. found that concomitant PPI use did not significantly reduce PFS or OS in patients receiving palbociclib.^22^ In contrast to palbociclib, some studies suggest that ribociclib’s bioavailability is less susceptible to PPIs. A study found no statistical difference in PFS between ribociclib users with and without concomitant PPIs (35.3 months vs 49.2 months, *P* = 0.594).^23^ Another retrospective study comparing PPI users and non-users among patients taking palbociclib or ribociclib also found no significant difference in PFS in ribociclib but shorter PFS in palbociclib. This suggests that ribociclib might be a preferred CDK4/6i in patients requiring concomitant PPI therapy.^24^ However, the study in rats revealed the transport, absorption, and plasma concentration of palbociclib and ribociclib was significantly suppressed by the administration of omeprazole, and rabeprazole.^25^ Meanwhile, the impact of PPIs on abemaciclib is less clear compared with palbociclib and ribociclib. Only Takahashi’s study found that concomitant PPI use did not significantly reduce PFS or OS in patients receiving abemaciclib.^22^ Some meta-analyses have indicated a potential adverse effect of PPIs on ribociclib as well, warranting careful consideration. A comprehensive meta-analysis incorporating ten research articles found a notable reduction in OS among patients receiving CDK4/6i who also used PPIs (HR = 2.00; 95% CI, 1.35-2.96). Nevertheless, the meta-analysis did not find a substantial correlation between PPI use and PFS of patients (HR = 1.30; 95% CI, 0.98-1.74).^26^ Another meta-analysis indicated that the simultaneous use of PPIs with palbociclib or ribociclib may be both associated with a shorter PFS in HR+, HER2− metastatic breast cancer patients.^27^ These results were inconsistent due to the high heterogeneity of the study’s design. For instance, Marzia Del Re’s study defined “no concomitant PPIs” as no PPIs use, but only administration of PPIs covered the entire or not less than 2/3 of treatment with CDK4/6i was defined as “concomitant PPIs.” Additionally, the study for ribociclib concomitant with PPIs in 2022 only included patients who were previous users of PPIs and excluded those given PPIs after initiating ribociclib. On the other hand, Takahashi’s study defined “no concomitant use of PPIs” as PPI administration covered the duration of less than ½ of CDK4/6i treatment, and “concomitant use of PPIs” was defined as covering more or equal to ½ of treatment. These differences may lead to misclassification bias and reduce comparability across studies. Our study found that no matter the administration of PPIs covered 10%, 10-50%, or more than 50% of CDK4/6i treatment, all significantly compromised PFS and OS. Moreover, more concomitant PPIs use worsens the outcomes. Therefore, if the cut point of concomitant use of PPIs was set at ½ or 2/3, the impact of PPIs in the CDK4/6i efficacy may be diminished, causing inconsistent study results.

There have been several proposed mechanisms for the interaction between PPIs and certain CDK4/6i. Studies have shown that increasing gastric pH with PPIs can reduce palbociclib exposure. Palbociclib and ribociclib are weak base drugs, and their solubility decreases in less acidic environments. According to the labels for palbociclib and ribociclib, the pKa values are approximately 3.9-7.4 and 5.3-8.5, respectively. The solubility of palbociclib in aqueous media decreases over the pH range of 4.3 to 9.0, dropping from greater than 0.7 mg/mL to less than 0.002 mg/mL. In ribociclib, the solubility decreases to 0.8 mg/mL at pH 6.8, compared with more than 2.4 mg/mL in acidic solutions. PPIs irreversibly inhibit the H+/K+-ATPase pump, leading to a significant increase in gastric pH, which raises it from 2.0 to over 6.0, thereby impairing the dissolution and subsequent absorption of these weak base drugs in the stomach.^28^ Specifically, the co-administration of rabeprazole with palbociclib resulted in a substantial decrease in palbociclib’s area under curve (AUC) by 62% and Cmax by 80% under fasting conditions. Even in a fed state, a reduction of 13% in AUC and 41% in maximum concentration (Cmax) was observed.^29^ This reduction in palbociclib bioavailability could potentially to compromise its therapeutic efficacy. However, a previous study showed gastric pH changes do not affect ribociclib and abemaciclib bioavailability.^30–32^ Although generally to a lesser extent than PPIs, it is important to note that H2-blockers also reduce gastric acidity. These agents are thought to have a similar but more minor impact on the absorption of pH-sensitive drugs like palbociclib compared with PPIs. If the co-administration of PPIs demonstrates a significant effect on drug exposure, further evaluation of the impact of H2-blocking agents and antacids would be warranted.^30^

The potential for drug–drug interactions between PPIs and CDK4/6i is possibly mediated through their effects on CYP3A4. Among the CDK4/6i, palbociclib is a weak time-dependent inhibitor of CYP3A4. Its heavy reliance on CYP3A for metabolism makes palbociclib susceptible to interactions with drugs that affect CYP3A activity.^33^^,^^34^ Ribociclib is a moderate to strong inhibitor of CYP3A4, but not induce CYP3A4 in vitro.^35^ Abemaciclib is a sensitive substrate of CYP3A4 but does not appear to significantly inhibit or induce this enzyme.^36^ In terms of PPIs, all of four PPIs, lansoprazole, pantoprazole, esomeprazole, and rabeprazole, undergo extensive hepatic metabolism primarily via CYP2C19 with minor metabolic pathways involve CYP3A4.^37^ While none of the common PPIs are considered strong inhibitors of CYP3A4, some exhibit weak to moderate inhibitory effects.^38^ When a PPI inhibits the activity of CYP3A4, the co-administration of PPI and CDK4/6i can lead to reduced metabolism and higher expected plasma concentrations of the CDK4/6i. This effect can have significant clinical consequences, primarily related to increased toxicity. For instance, ribociclib is known to cause QTc interval prolongation. Similarly, increased levels of palbociclib or abemaciclib could lead to a higher incidence or severity of their common side effects, such as neutropenia, fatigue, or gastrointestinal disturbances. In our study, we also noted patients had a higher proportion of grade 3 neutropenia and creatinine increased in concomitant use of PPIs, which is compatible with the thesis that PPI inhibits the activity of CYP3A4 causing increased toxicity of CDK4/6i. However, several retrospective studies suggest that PPI use may also be associated with elevated serum creatinine.^39^^,^^40^ This is an important consideration for future evaluations to differentiate changes in kidney function related to CDK4/6i treatment, PPI use, or a drug–drug interaction between the two.^41^

The efficacy of oral anticancer medications, including hormone therapy, CDK4/6i, and metronomic therapy, is influenced by both absorption and drug–drug interactions, as well as medication adherence. Real-world studies have demonstrated a wide range of adherence rates, from 26%-79% for hormone therapy and 17%-90% for other oral systemic anticancer therapies, varying by medication type and treatment duration.^42–44^ A cross-­sectional study also revealed that metastatic breast cancer patients displayed significantly higher adherence than non-metastatic patients.^45^ A significant limitation of retrospective studies is the difficulty in accurately assessing treatment compliance, which is typically estimated from prescription refill data and electronic health records. This study lacks precise information regarding the compliance of CDK4/6i, hormone therapy, or antacid treatments. An alternative method to gauge compliance is to compare the adverse effects and outcomes of the entire cohort with those of clinical trials, which typically feature precise treatment protocols and monitoring to ensure adherence. For example, a previous study from our institution found that the rates of adverse effects and outcomes were comparable with those reported in CDK4/6i clinical trials, suggesting that medication compliance in our cohort was likely acceptable.^46^

A significant difference in the Charlson Comorbidity Index (CCI) was observed between the groups in this study. The CCI, derived from ICD-9 and ICD-10 codes, includes 19 items. In our cohort of patients with metastatic breast cancer, the majority had a CCI score of at least 8, as a malignancy is weighted with 2 points and a metastatic solid tumor with 6 points. Furthermore, patients prescribed PPIs or H2-blockers under the national health insurance system must have documented peptic ulcer disease, which adds 1 point to their CCI score. Therefore, patients using antacids were more likely to have a higher CCI. In the multivariate Cox proportional hazards model, the proportion of PPI use during CDK4/6i treatment remained a significant independent risk factor for disease progression, whereas the CCI did not. However, in the multivariate Cox proportional hazards model for mortality, both the proportion of PPI use and the CCI were significant independent risk factors. A higher comorbidity score is associated with an increased risk of mortality, which makes it difficult to determine whether the poorer outcomes are primarily due to PPI use or the patient’s underlying health status.

Understanding the potential interactions of CDK4/6i with concomitant medications like PPIs is crucial for optimizing patient outcomes. Especially, prescribing information on drug–drug interactions tends to focus on metabolizing enzymes, such as the cytochrome P450 superfamily, rather than the effect of acid-reducing therapy on absorption mechanisms. This study gives real-world evidence of the co-administration of PPIs and H2-blockers with 3 types of CDK4/6i, including the dosage impact of PPIs. Nevertheless, this study had some limitations. Firstly, owing to the retrospective database from a single-center study, some vital factors such as comorbidities and patients’ subjective adverse effects may be missing. Additionally, the information on antacids or other CYP3A4 inhibitors or inducers from other facilities was not included in the data. These confounding factors affect the analysis of disease outcomes. Secondly, there was limited sample size and follow-up time, especially in abemaciclib, which is not feasible to determine overall survival. Finally, the concurrent use of antacids with CDK4/6i in patients with HR+ metastatic breast cancer presents a complex clinical scenario; for instance, the washout period of antacids and intermittent use of antacids should be considered as time variates. To more accurately identify the subgroup of patients with poor outcomes, future prospective studies in real-world settings should incorporate a more precise assessment of patient medication compliance. Additionally, these studies should systematically document the use of antacids and other cytochrome P450 3A4 (CYP3A4) inhibitors or inducers. It is also essential to appropriately adjust for patient comorbidities to better isolate the effects of the treatments. While there are inconsistent results of studies on this issue, some meta-analyses suggest a potential negative impact of PPIs on treatment efficacy in patients receiving CDK4/6i. Clinicians should exercise caution when co-prescribing antacids with CDK4/6i, particularly PPIs. Further prospective research is needed to provide more definitive guidance on managing this common co-prescription to optimize treatment outcomes for patients with HR+ metastatic breast cancer.

## Conclusion

The use of PPIs, and to a lesser extent H2 blockers, in combination with CDK4/6i and endocrine therapy is associated with adverse effects on PFS and OS in patients with HR+ metastatic or recurrent breast cancer in a real-world setting. Clinicians should exercise caution when prescribing proton pump inhibitors to patients undergoing CDK4/6i therapy.

## Supplementary Material

oyaf268_Supplementary_Data

## Data Availability

The data underlying this article cannot be shared publicly due to reasons of personal privacy. The data will be shared on reasonable request to the corresponding author.
