# Supplementary figures and images for: Real-world evaluation of CDK4/6 inhibitors in hormone receptor-positive metastatic breast cancer: prognostic effect of proton pump inhibitor use
Source: Oncologist. 2025 Sep 2;30(10):oyaf268. doi: 10.1093/oncolo/oyaf268 (PMC12505142; doi:10.1093/oncolo/oyaf268)

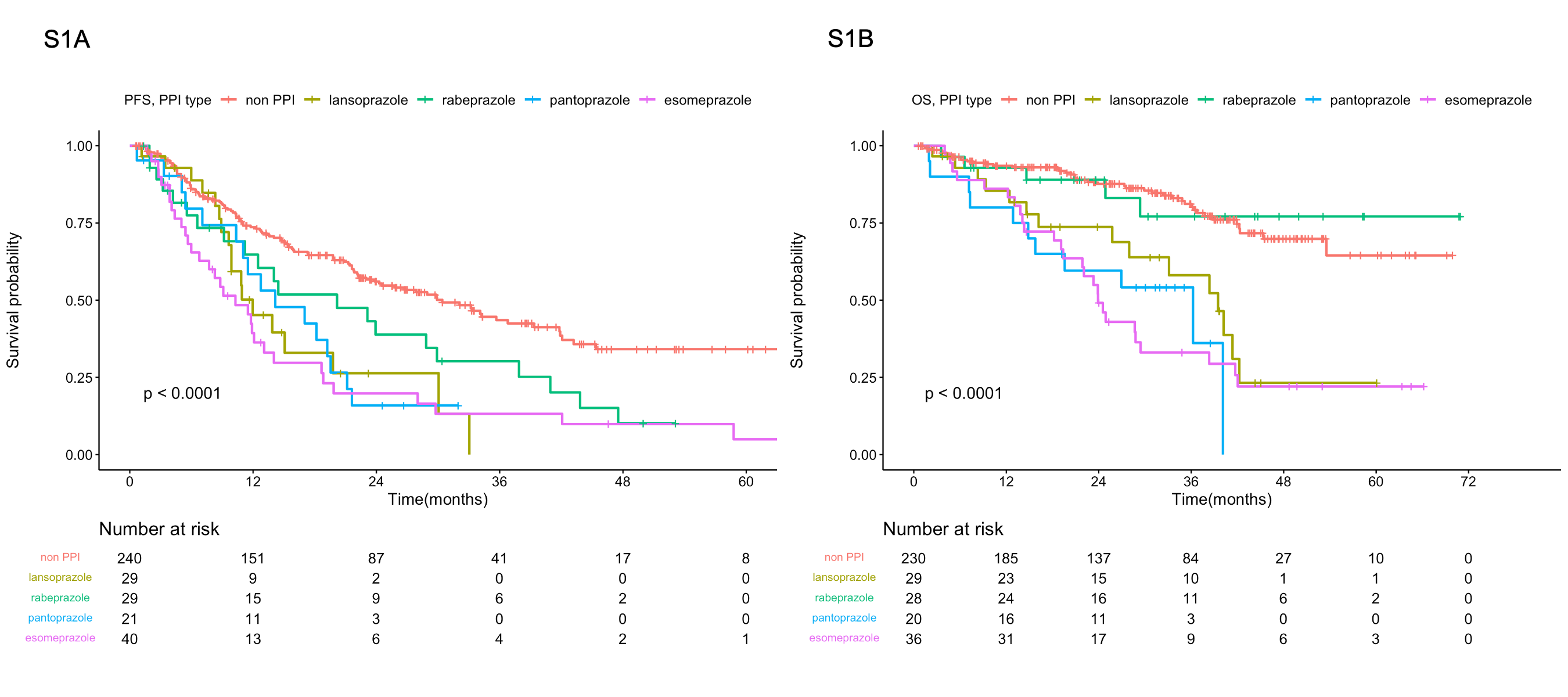

Supplement: oyaf268_Supplementary_Data [file oyaf268_supplementary_data.zip › FigS1.tiff]

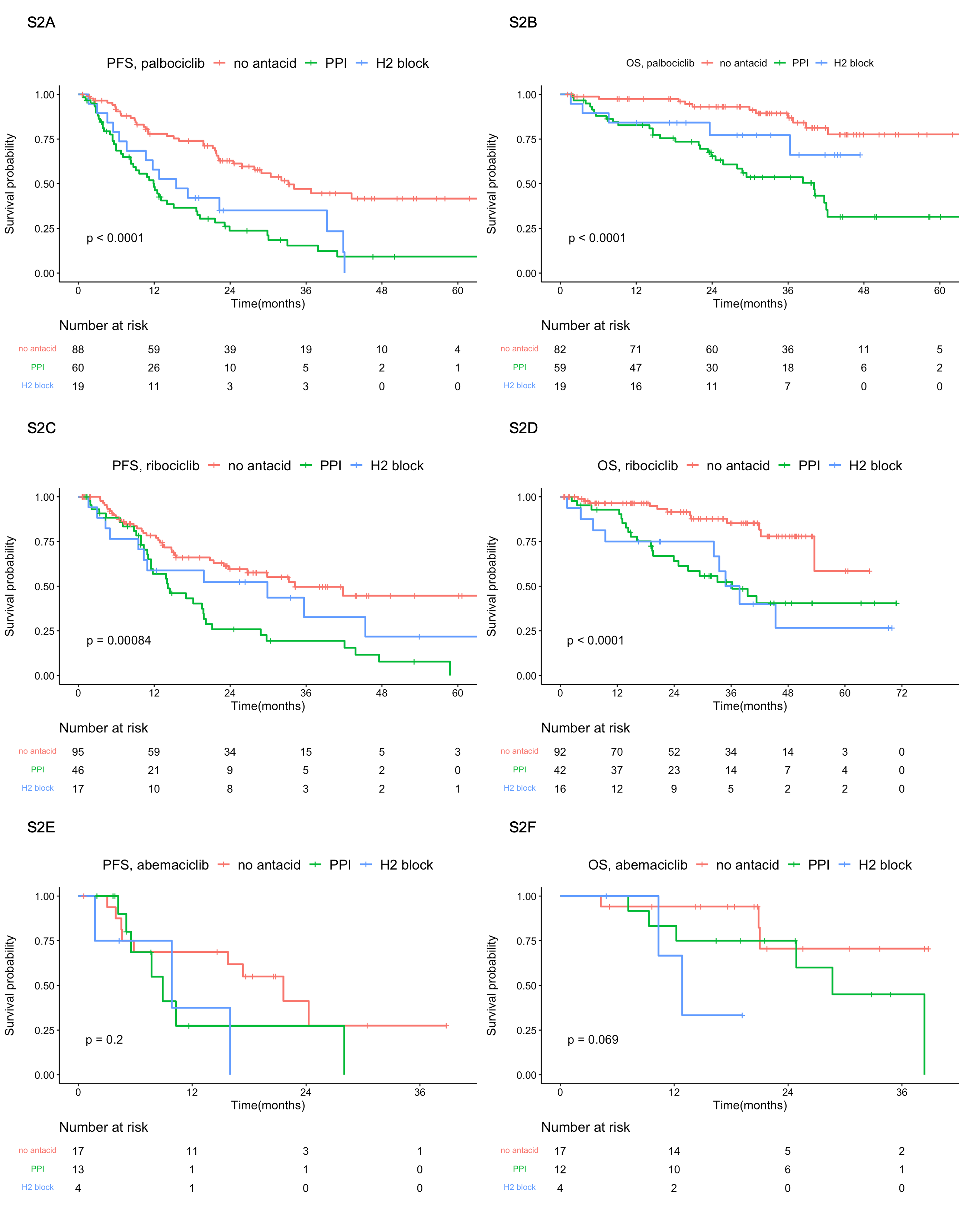

Supplement: oyaf268_Supplementary_Data [file oyaf268_supplementary_data.zip › FigS2.tiff]
