# Supplementary material for: Real-world evaluation of CDK4/6 inhibitors in hormone receptor-positive metastatic breast cancer: prognostic effect of proton pump inhibitor use
Source: Oncologist. 2025 Sep 2;30(10):oyaf268. doi: 10.1093/oncolo/oyaf268 (PMC12505142; doi:10.1093/oncolo/oyaf268)
Supplement: oyaf268_Supplementary_Data [file oyaf268_supplementary_data.zip › Supplement Fig legends.doc]

**Supplemental Figure legends**

**Fig S1 Kaplan–Meier survival curve analyses for PFS(S1A) and OS(S1B) among patients treated with CDK4/6 inhibitors without antacids, and with 4 types of PPIs, lansoprazole, rabeprazole, pantoprazole and esomeprazole.**

**Fig S2 Kaplan–Meier survival curve analyses for PFSand OS among patients treated with 3 types of CDK4/6 inhibitors without antacids, with PPIs, or with H2 blockers only.** (S2A) PFS and (S2B) OS in the palbociclib cohort. (S2C) PFS and (S2D) OS in the ribociclib cohort. (S2E) PFS and (S2F) OS in the abemaciclib cohort.
